# SPINK1 facilitates tumor progression via the EGFR/JAK/STAT3 axis in oral squamous cell carcinoma: insights from single-cell RNA sequencing

**DOI:** 10.3389/fonc.2025.1585277

**Published:** 2025-08-19

**Authors:** Mingyan Bao, Zhangui Tang

**Affiliations:** Hunan Key Laboratory of Oral Health Research & Hunan 3D Printing Engineering Research Center of Oral Care & Hunan Clinical Research Center of Oral Major Diseases and Oral Health & Academician Workstation for Oral-maxilofacial and Regenerative Medicine & Xiangya Stomatological Hospital & Xiangya School of Stomatology, Central South University, Changsha, Hunan, China

**Keywords:** SPINK1, OSCC, single-cell RNA sequencing, EGFR, JAK/STAT3 signaling pathways

## Abstract

**Objective:**

This study aimed to elucidate the functional role and molecular mechanisms of Serine Peptidase Inhibitor Kazal Type 1 (SPINK1) in oral squamous cell carcinoma (OSCC) through integrative analysis of single-cell RNA sequencing (scRNA-seq) data.

**Materials and methods:**

Cellular subpopulations within OSCC were stratified using transcriptomic datasets from the GEO database. Cell-cell communication networks were reconstructed to map ligand-receptor interactions, while Gene Set Variation Analysis (GSVA) and Gene Set Enrichment Analysis (GSEA) were employed to systematically investigate SPINK1-associated signaling pathways. SPINK1 expression profiles in OSCC tissues were validated through quantitative PCR (qPCR) and immunoblotting. Gain- and loss-of-function assays utilizing Cell Counting Kit-8 (CCK-8), wound healing assays, transwell migration/invasion chambers, and murine xenograft models were implemented to assess SPINK1-mediated oncogenic phenotypes. Rescue experiments conclusively established the EGFR/JAK/STAT3 signaling axis as the mechanistic backbone of SPINK1-driven oncogenesis.

**Results:**

SPINK1 was closely associated with T cells, malignant cells, and an array of immune modulators, including chemokines and immunoinhibitors, throughout OSCC progression. SPINK1 operates through pathways involving JAK/STAT3, P53, Notch and WNT signaling cascades. Relative to their normal tissue counterparts, SPINK1 is upregulated in OSCC, resulting in increased cell proliferation, invasion, and migration upon SPINK1 overexpression, whereas SPINK1 knockdown has opposite effects. SPINK1 knockdown led to a significant reduction in EGFR and STAT3 phosphorylation levels, whereas exogenous supplementation of EGFR effectively rescued this phenotype.

**Conclusion:**

SPINK1 has been established as a novel therapeutic target in OSCC, with its dual role in tumorigenesis and immune modulation providing a molecular foundation for developing targeted therapeutic modalities and precision oncology strategies.

## 1 Introduction

Oral squamous cell carcinoma (OSCC) constitutes over 90% of oral malignancies and ranks as the 18th most prevalent cancer worldwide, with a persistently poor 5-year survival rate of 50%-60%, despite diagnostic and therapeutic advancements (1–3), his clinical challenge stems from its aggressive biological behavior, characterized by local tissue infiltration (observed in 43% of T3/T4 tumors) and cervical lymph node metastasis (occurring in 38% of initially node-negative cases) (4, 5) or patients with advanced oral cancer (clinical stage III/IVA), even after receiving comprehensive surgical management and postoperative chemoradio therapy, the median survival time rarely exceeds 30 months (6, 7), despite advancements in treatment, the prognosis for these patients has not significantly improved over the past decade (8–10). Onsequently, the identification of novel therapeutic targets represents a critical imperative in improving clinical outcomes for these patients.

Serine protease inhibitor Kazal type 1 (SPINK1), a member of the Kazal-type serine protease inhibitor family, was initially identified in the urine of ovarian cancer patients (11, 12), it is secreted by pancreatic acinar cells into the pancreatic duct, where it inhibits trypsin activity. The SPINK1 gene is located on chromosome 5q32 of the human genome and features a compact structure composed of 4 exons and 3 introns (13).

Recent studies have revealed that SPINK1 is closely associated with malignant progression in various solid tumors and correlates significantly with patient prognosis. In hepatocellular carcinoma, SPINK1 promotes tumor proliferation and serves as a prognostic marker (14). In prostate cancer, urinary SPINK1 levels increase with advancing Gleason score, pathological T stage, and metastatic status, demonstrating significant predictive value for patient prognosis (15). SPINK1 also functions as a prognostic marker in colorectal cancer, gastric cancer, and renal cell carcinoma (16–18), with differential expression observed across multiple solid tumors, including but not limited to ovarian cancer, bladder cancer, gallbladder cancer, lung cancer, and breast cancer (19–22). Notably, SPINK1 shows promise as an early diagnostic biomarker: its concentration increases by 1000-fold in malignant ovarian tumors (23, 24), and it exhibits diagnostic relevance in both hepatocellular and pancreatic malignancies (25, 26).

Mechanistically, SPINK1 primarily mediates tumor progression through two pathways:As a member of the serine protease inhibitor family, SPINK1 disrupts the protease-antiprotease balance, leading to abnormal degradation of the extracellular matrix. Additionally, its structural similarity to EGF enables it to bind and activate EGFR downstream signaling pathways, triggering epithelial-mesenchymal transition (EMT) and enhancing tumor cell metastatic potential (27, 28). Second, SPINK1 induces stromal cells to secrete immune factors, sustaining the activation of pro-inflammatory cytokine networks. Studies in colorectal cancer models demonstrate that SPINK1 enhances STAT3 phosphorylation in an IL-6-dependent manner, thereby promoting tumor cell invasion and metastasis (29, 30). This suggests that SPINK1 may drive malignant progression through similar inflammatory mechanisms in other cancers.

However, research on SPINK1 in head and neck tumors remains limited. Early studies confirmed elevated serum SPINK1 levels in head and neck squamous cell carcinoma patients, positively correlating with disease progression from normal tissue to inflammatory lesions, benign tumors, and ultimately malignancies (31, 32). Bioinformatics analyses further highlight its strong prognostic association with tongue squamous cell carcinoma (33). SPINK1 also demonstrates potential as a biomarker for esophageal cancer (34). Thus, investigating SPINK1’s role and mechanisms in OSCC not only addresses critical gaps in understanding the pathogenesis of oral squamous cell carcinoma but also provides a theoretical foundation for developing novel therapeutic targets.

Single-cell RNA sequencing (scRNA-seq) has emerged as a transformative tool in OSCC research, enabling high-resolution mapping of tumor heterogeneity and stromal interactions (35). Pioneering studies have leveraged this technology to identify metastasis-initiating cell subpopulations and immunosuppressive niches specific to oral carcinogenesis (36). Intriguingly, while SPINK1 expression has been profiled in bulk tissue analyses, its cell type-specific regulation remains uncharacterized at single-cell resolution (37).

In this study, we provide a novel and comprehensive exploration of the mechanistic role of SPINK1 in OSCC, distinctively leveraging scRNA-seq to resolve tumor heterogeneity and cell-type-specific interactions within the tumor microenvironment, revealing SPINK1`s dual role in oncogenesis and immune modulation. We further conducted *in vivo* functional validation using murine xenograft models, demonstrating SPINK1`s causal role in tumor growth and metastasis—a critical advancement beyond correlative analyses. Most significantly, we elucidated the EGFR/JAK/STAT3 signaling axis as SPINK1`s core mechanistic pathway through gain/loss-of-function and rescue experiments, positioning SPINK1 upstream of this druggable cascade and offering a molecular basis for targeted therapies. These findings may contribute to improving clinical therapeutic strategies for OSCC and provide novel biomarkers for its treatment.

## 2 Method

### 2.1 Data acquisition

The study protocol was approved by the Institutional Review Board of Hunan Xiangya Stomatological Hospital, Central South University (Approval No. 20240030). Gene expression profiles of 12 OSCC gingival and buccal tissue samples were retrieved from the GSE215403 (38) dataset in the Gene Expression Omnibus (GEO) database (https://www.ncbi.nlm.nih.gov/geo/), a curated repository for high-throughput functional genomics data established by the National Center for Biotechnology Information (NCBI).

### 2.2 Single-cell transcriptomic analysis

Raw expression data were processed using the Seurat package. Low-quality cells were filtered (nFeature_RNA > 200, percent.mt < 10%, nCount_RNA < 200,000), followed by data normalization, variance stabilization, and principal component analysis (PCA). Harmony batch correction and UMAP dimensionality reduction were applied to cluster cells. Marker genes for each cluster were identified via differential expression analysis (FindAllMarkers function, logfc.threshold = 1).

### 2.3 Intercellular communication inference

Cell-cell signaling networks were reconstructed using CellCall, which integrates ligand-receptor interactions and transcription factor activity based on KEGG pathway-annotated ligand-receptor-transcription factor (L-R-TF) axes.

### 2.4 GSVA (gene set difference analysis)

Gene set variation analysis (GSVA) is a nonparametric, unsupervised method for assessing gene set enrichment. GSVA converts gene-level changes into pathway-level changes by comprehensively scoring the gene set of interest and then determining the biological function of the sample. In this study, gene sets were downloaded from the Molecular Signatures Database, and the GSVA algorithm was used to score each gene set comprehensively to evaluate potential biological function changes in different samples.

### 2.5 GSEA

Patients were stratified into SPINK1-high and SPINK1-low groups. Pathway divergence was assessed using GSEA with version 7.0 annotated gene sets from MSigDB. Significance thresholds were set as nominal p < 0.01 and false discovery rate (FDR) q < 0.25.

### 2.6 Cell lines, cell culture conditions, and transfection

Human oral keratinocytes (HOKs) and the OSCC cell lines SCC9, SCC25, HN4, HN30, and CAL27 were procured from the China Center for Type Culture Collection. HOKs were cultured in keratinocyte medium and OSCC cell lines cells were cultured in Dulbecco’s modified Eagle’s medium (DMEM; Biological Industries, Israel, C3120-0500) with 10% heat-inactivated fetal bovine serum (Biological Industries, Israel, 04-001-1C), 100 units/mL penicillin, and 100 g/mL streptomycin, maintained at 37°C in a humidified 5% CO2 atmosphere.

The shSPINK1 plasmid (GeneCopoeia, China, XM_017009906) was constructed using the GV248 vector. A SPINK1 overexpression vector (GV658, CMV enhancer-MCS-polyA-EF1A-zsGreen-sv40-puromycin) was created based on the SPINK1 reference sequence (NM_001379610.1) from the National Biotechnology Information Center database. Transient transfection was performed using Lipofectamine 3000 (Invitrogen, USA, L3000015) following the manufacturer’s instructions. The transfected cells were cultured and harvested for subsequent assays.

### 2.7 RNA extraction and qRT-PCR

Total RNA was isolated using TRIzol Reagent, followed by cDNA synthesis with HiScript II Q RT SuperMix (Vazyme). qRT-PCR was performed using ChamQ Universal SYBR qPCR Master Mix (Vazyme) with GAPDH as the endogenous control.

SPINK1 primers:

F:5’-TCTATCTGGTAACACTGGAGCTG-3’,

R:5’-ACACGCATTCATTGGGATAAGT-3’.

GAPDH primers:

F:5’-GGAGTCCACTGGCGTCTTCA -3’,

R:5’-GTCATGAGTCCTTCCACGATACC -3’.

shSPINK1 primers:

ShSPINK1-1:5’-GCCAAATGTTACAATGAACTT-3’,

ShSPINK1-2:5’-CCAATGAATGCGTGTTATGTT-3’,

ShSPINK1-3:5’-CCCTGTTGAGTCTATCTGGTA-3’.

EGFR primers:

F:5’-AGGCACGAGTAACAAGCTCAC -3’

R:5’-ATGAGGACATAACCAGCCACC -3’

JAK primers:

F:5’-ATCCACCCAACCATGTCTTCC -3’

R:5’-ATTCCATGCCGATAGGCTCTG -3’

STAT3 primers:

F:5’-ACCAGCAGTATAGCCGCTTC -3’

R:5’-GCCACAATCCGGGCAATCT -3’

### 2.8 Western blotting

Cells at 80–90% confluence were lysed, and proteins (30–40 μg) were resolved on 6% or 8% SDS-PAGE and transferred to PVDF membranes (Merck). After blocking with 4% BSA, membranes were incubated overnight at 4°C with primary antibodies: anti-SPINK1 (Bioss, 1:1000) and anti-GAPDH (Bioss, 1:1000). IRDye-labeled secondary antibodies (Bioss, 1:10,000) were used, and signals were visualized using an Odyssey Imaging System.

### 2.9 Cell proliferation analysis

SPINK1-transfected cells (1,000 cells/well in 96-well plates) were analyzed using Cell Counting Kit-8. Absorbance was measured at 0, 12, 24, 48, and 72 h using a microplate reader.

### 2.10 Wound-healing assay

Confluent cells in 6-well plates were serum-starved for 24 h, scratched with a 10 μL pipette tip, and washed. Migration was monitored in serum-free medium, with wound closure quantified at 24 h using ImageJ v1.54p software.

### 2.11 Transwell migration and invasion assay

For Transwell migration assay: Confluent cells were resuspended in serum-free medium to a density of 1×10^5 cells/ml. 200 μl cell suspension was added to the upper chamber, while 600 μl medium containing 10% FBS was loaded into the lower chamber. After 24-hour incubation, cells were fixed with 4% paraformaldehyde for 30 minutes and stained with 0.1% crystal violet for 15 minutes. Quantitative analysis was performed using ImageJ v1.54p software. For Transwell invasion assay: Matrigel was diluted with serum-free DMEM to 250 μg/ml. 50 μl diluted Matrigel was coated onto each Transwell insert and polymerized at 37°C for 1 hour. Subsequent procedures followed the same protocol as the migration assay.

### 2.12 Animal assays

BALB/c nude mice (4-week-old, n=6/group) were subcutaneously injected with 1×10^7^ shNC-CAL27 or shSPINK1-CAL27 cells. Tumor volume was measured every 3 days (volume = ½ × [width² × length]). Mice were euthanized at 3 weeks for tumor excision and weighing.

### 2.13 Statistical analysis

GEO data were analyzed using R (v4.2.2; p<0.05). Experimental data were processed with GraphPad Prism 9.3.0. Two-group comparisons at different time points used Two-way ANOVA. Two-group comparisons used Student’s t-test (p<0.05).

## 3 Results

### 3.1 Single-cell sample subtype cluster analysis

The GSE215403 dataset downloaded from the NCBI GEO public database contains 12 gingival and buccal tissue samples of oral squamous cell carcinoma. This analysis revealed the expression of SPINK1 in the original data of GSE215403 (**Supplementary Figure S1A**). Only cells with nFeature_RNA > 200 & percent.mt < 10 & nCount_RNA < 200000 in the expression profile were subsequently retained for this analysis, for a total of 34,297 cells. The feature expression levels were included for subsequent analysis (**Supplementary Figures S1B, C**). The 10 genes with the highest standard deviations are displayed in **Supplementary Figure S1D**.

**Figure 1:**
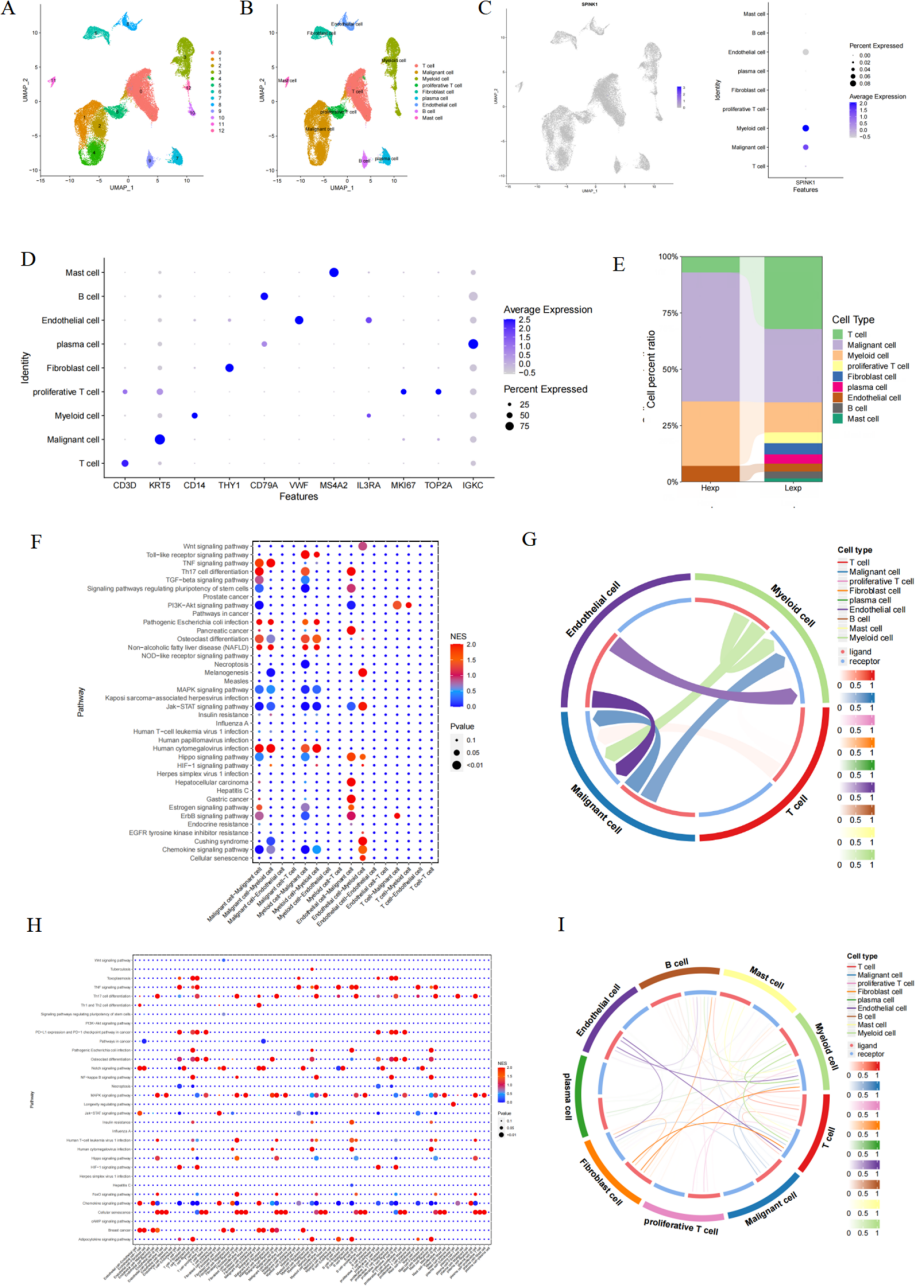
**(A)** OSCC cells were divided into 13 cell clusters; **(B)** Cell clusters were typed; **(C)** Expression of SPINK1 in cell clusters; **(D)** Expression of markers in each cell type; **(E)** Proportion of cell types in Hexp/Lexp cell; **(F, G)** Cell communication types with high SPINK1 expression; **(H, I)** Cell communication types with low SPINK1 expression.

In this study, 2000 hypervariable genes were selected, and PCA dimensionality reduction analysis was subsequently conducted to determine that there was a batch effect among samples (**Supplementary Figure S1E**). Harmony analysis was further used to reduce dimensionality to eliminate batches (**Supplementary Figure S1F**). The optimal number of pcs was 30 according to ElbowPlot (**Supplementary Figure S1G**), and 13 cell subpopulations were obtained via UMAP analysis at 0.2 resolution (**Figure 1A**). Annotation based on canonical marker expression patterns classified these clusters into nine major cell types: T cells, malignant cells, myeloid cells, proliferative T cells, fibroblasts, plasma cells, endothelial cells, B cells, and mast cells (**Figures 1B, D**). We further quantified the proportional distribution of these cell types between SPINK1-high and SPINK1-low expression groups (**Figure 1E**).

### 3.2 SPINK1 expression in single-cell data and cell-call communication analysis

SPINK1 exhibited heterogeneous expression patterns across the nine annotated cell types (**Figure 1C**). To investigate its functional relevance, we performed ligand-receptor interaction analysis using CellCall on SPINK1-high and SPINK1-low cells. Bubble plots revealed distinct pathway activation profiles: SPINK1-high cells showed enhanced activity in chemokine signaling, Jak-STAT signaling, and osteoclast differentiation pathways, whereas SPINK1-low cells displayed predominant engagement of MAPK signaling, TNF signaling, and chemokine pathways. A circular interaction diagram further delineated the directionality and intensity of intercellular communication mediated by specific ligand-receptor pairs.(**Figures 1F–I**).

### 3.3 Analysis of the correlation between SPINK1 and immune factors

**Figure 2:**
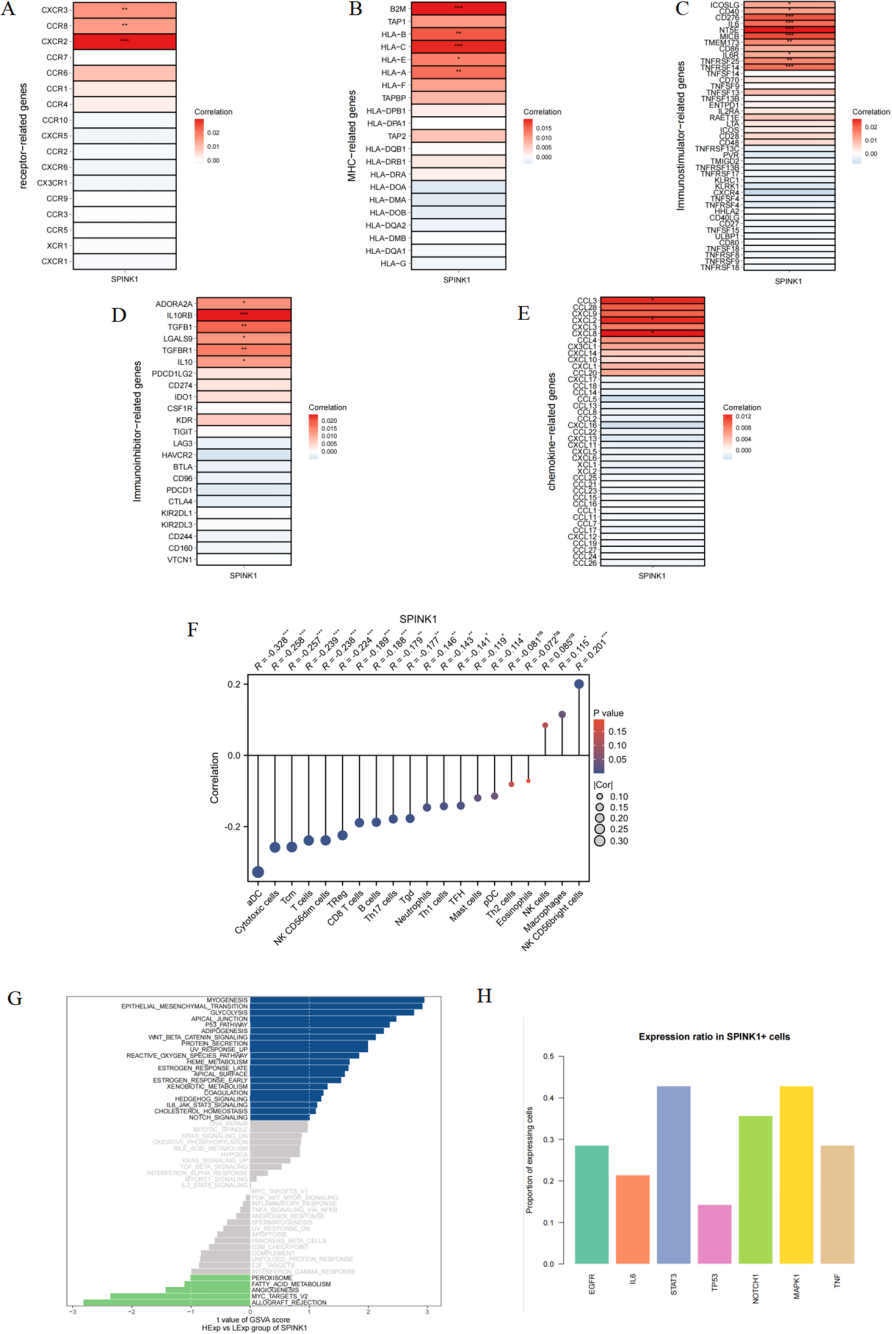
**(A–E)** Correlation of SPINK1 with receptor, MHC, immunoinhibitors, immunostimulators, and chemokines; **(F)** Correlation of SPINK1 with immune cell infiltration levels; **(G)** GSVA analysis of SPINK1; **(H)** Expression rate of key factors in each pathway in SPINK1+ cells.

This study revealed a correlation between SPINK1 and different immune factors, including immune regulatory factors, chemokines, cell receptors, and human leukocyte antigens, from the TISIDB database. SPINK1 was strongly correlated with immune factors, among which chemokines were significantly positively correlated with CCL3, CXCL2, and CXCL8; immunoinhibitors were significantly positively correlated with ADORA2A, IL10RB, TGFB1, etc.; immunostimulators were significantly positively correlated with NT5E, MICB, and CD276; MHC was significantly positively correlated with B2M, HLA-C, and HLA−A; and CXCR2, CCR8, and CXCR3 in the receptor were significantly positively correlated with CXCR2, CCR8, and CXCR3 (**Figures 2A–E**). These analyses confirmed that these key genes are closely related to the level of immune cell infiltration and play important roles in the immune microenvironment.

### 3.4 SPINK1 immune infiltration analysis

This study investigated the correlation between SPINK1 expression and infiltration levels of 20 common immune cell types. The results revealed that SPINK1 was negatively correlated with 15 immune cell populations, including antigen-presenting cells (aDC) and cytotoxic immune subsets such as cytotoxic cells, Tcm, T cells, and NK CD56dim cells. Conversely, SPINK1 showed significant positive correlations with macrophages and NK CD56bright cells. These findings suggest that high SPINK1 expression may broadly suppress antitumor immune responses and facilitate tumor immune escape, potentially through mechanisms such as EGFR pathway activation and STAT3-mediated immunosuppressive signaling (**Figure 2F**).

### 3.5 Signaling pathways in which SPINK1 participates

Next, we will study the specific signaling pathways associated with SPINK1 and explore the potential molecular mechanisms by which SPINK1 affects the progression of oral squamous cell carcinoma. The GSVA results revealed that SPINK1 was enriched in P53 PATHWAY,NOTCH SIGNALING, WNT-BETA_CATENIN SIGNALING, IL6/JAK/STAT3 SIGNALING and other signaling pathways (**Figure 2G**). To further identify SPINK1-associated pathways, we analyzed the positivity rates of key molecules across multiple signaling pathways in SPINK1-positive cells. The results showed that STAT3, NOTCH1, MAPK1, and EGFR exhibited the highest correlation with SPINK1 expression (**Figure 2H**). These findings suggest that SPINK1 may affect the progression of oral squamous cell carcinoma through these pathways.

### 3.6 Activity differences and coexpression analysis between SPINK1 and metabolic pathways

**Figure 3:**
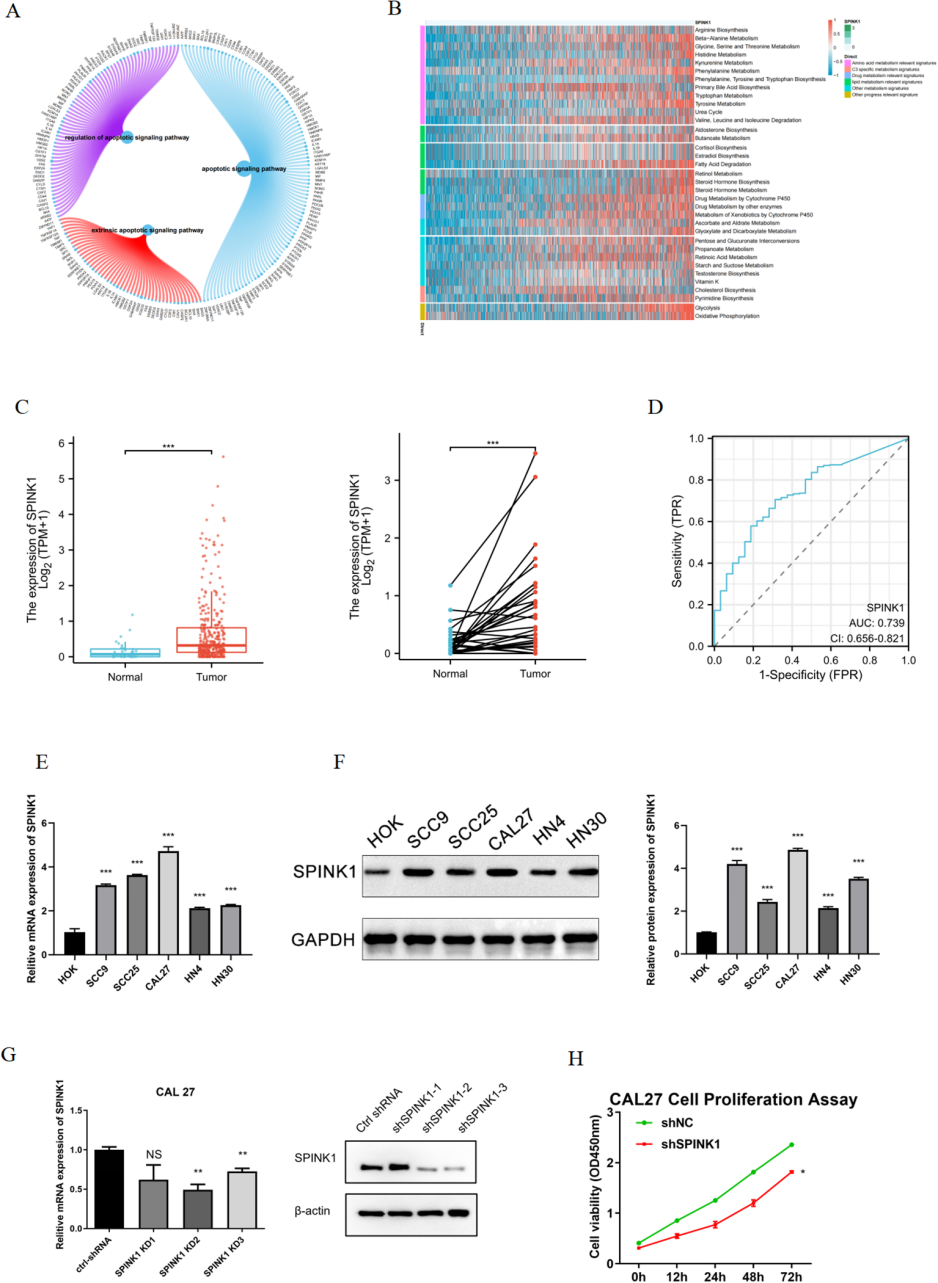
**(A)** Enrichment of SPINK1 in apoptosis-related pathways; **(B)** Enrichment of SPINK1 in metabolism-related pathways; **(C)** Expression of SPINK1 in TCGA; **(D)** Diagnostic efficacy of SPINK1; **(E)** Expression of SPINK1 mRNA in OSCC cells; **(F)** Expression of SPINK1 protein; **(G)** Knockdown efficiency of SPINK1; **(H)** CCK-8 assay after SPINK1 knockdown.

GSEA revealed that SPINK1 was enriched mainly in the apoptotic signaling pathway, extrinsic apoptotic signaling pathway, regulation of the apoptotic signaling pathway and other signaling pathways (**Figure 3A**). In addition, This study used the gsva function of the GSVA package to perform ssGSEA to calculate the enrichment scores of different metabolic pathways for each cell. These scores were subsequently normalized, and a heatmap was generated to display the enrichment scores of different cells. The results revealed that the enrichment score of amino acid metabolism pathways was greater (**Figure 3B**).

### 3.7 SPINK1 upregulation in OSCC

**Figure 4:**
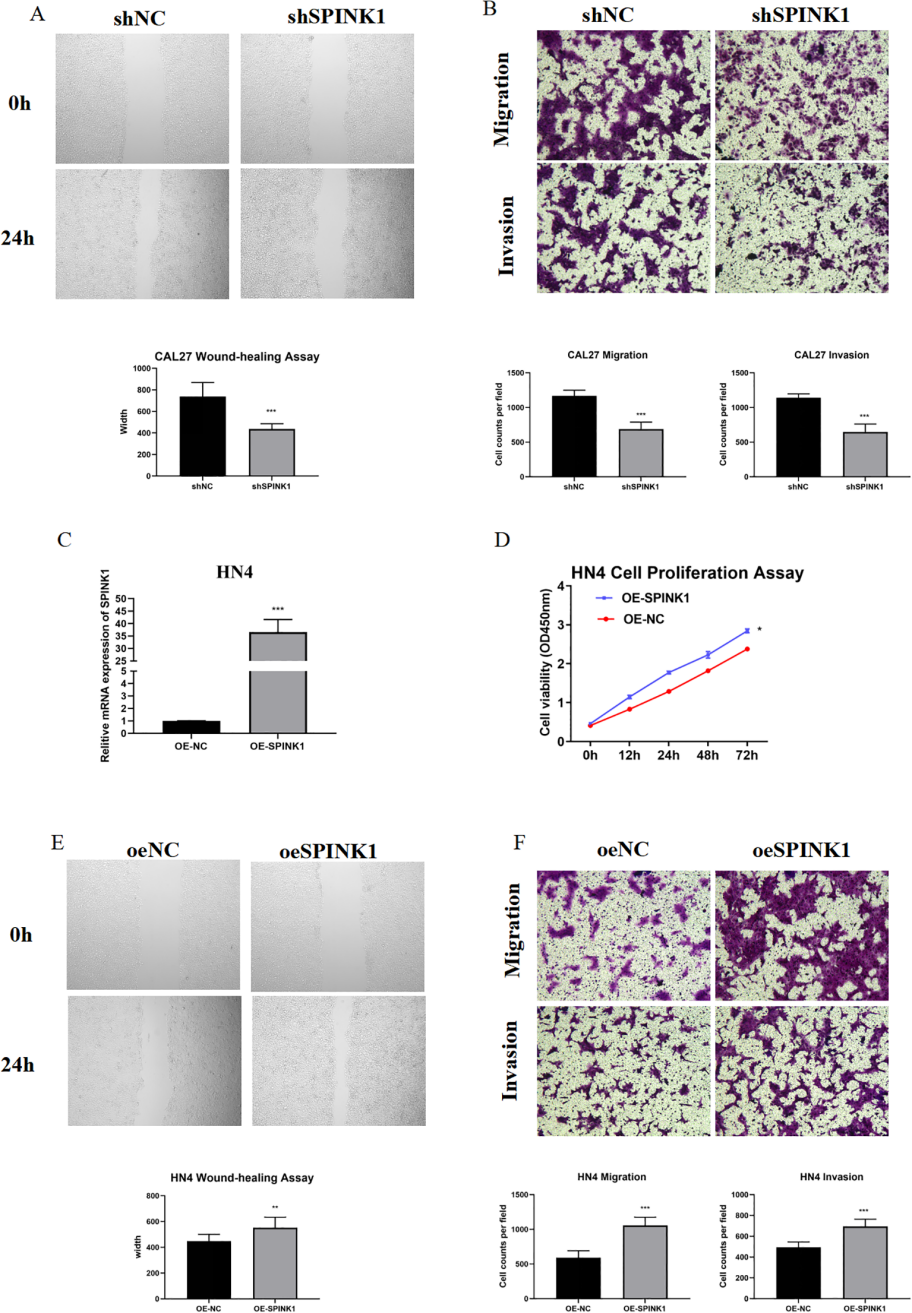
**(A)** Wound healing assay after SPINK1 knockdown; **(B)** Transwell assay after SPINK1 knockdown; **(C)** Overexpression efficiency of SPINK1; **(D)** CCK-8 assay after SPINK1 overexpression; **(E)** Wound healing assay after SPINK1 overexpression; **(F)** Transwell assay after SPINK1 overexpression.

This study validated SPINK1 expression in OSCC. TCGA data demonstrated elevated SPINK1 expression in OSCC tissues (**Figure 3C**) with robust diagnostic efficacy (AUC analysis, **Figure 3D**). qRT-PCR revealed significant SPINK1 mRNA upregulation in OSCC cell lines (SCC9, SCC25, CAL27, HN4, HN30) compared to normal oral keratinocytes (HOK) (**Figure 3E**). Western blot confirmed increased SPINK1 protein levels in OSCC cells (**Figure 4F**), aligning with its oncogenic roles in other cancers.

### 3.8 SPINK1 modulates OSCC biological behavior

CAL27 and HN4 cell lines were selected for functional assays based on SPINK1 expression levels. shRNA-mediated SPINK1 knockdown in CAL27 cells reduced mRNA expression by >50% (**Figure 3G**), accompanied by suppressed proliferation (CCK-8 assay, **Figure 3H**), impaired migration (40% reduction in wound healing, **Figure 4A**), and diminished invasion capacity (Transwell assay, **Figure 4B**). Conversely, after overexpression of SPINK1 (**Figure 4C**), the cell proliferation (**Figure 4D**), migration (wound healing assay, **Figure 4E**), and invasion (**Figure 4F**) of HN4 were significantly enhanced.

### 3.9 SPINK1 promotes *in vivo* tumor growth

**Figure 5:**
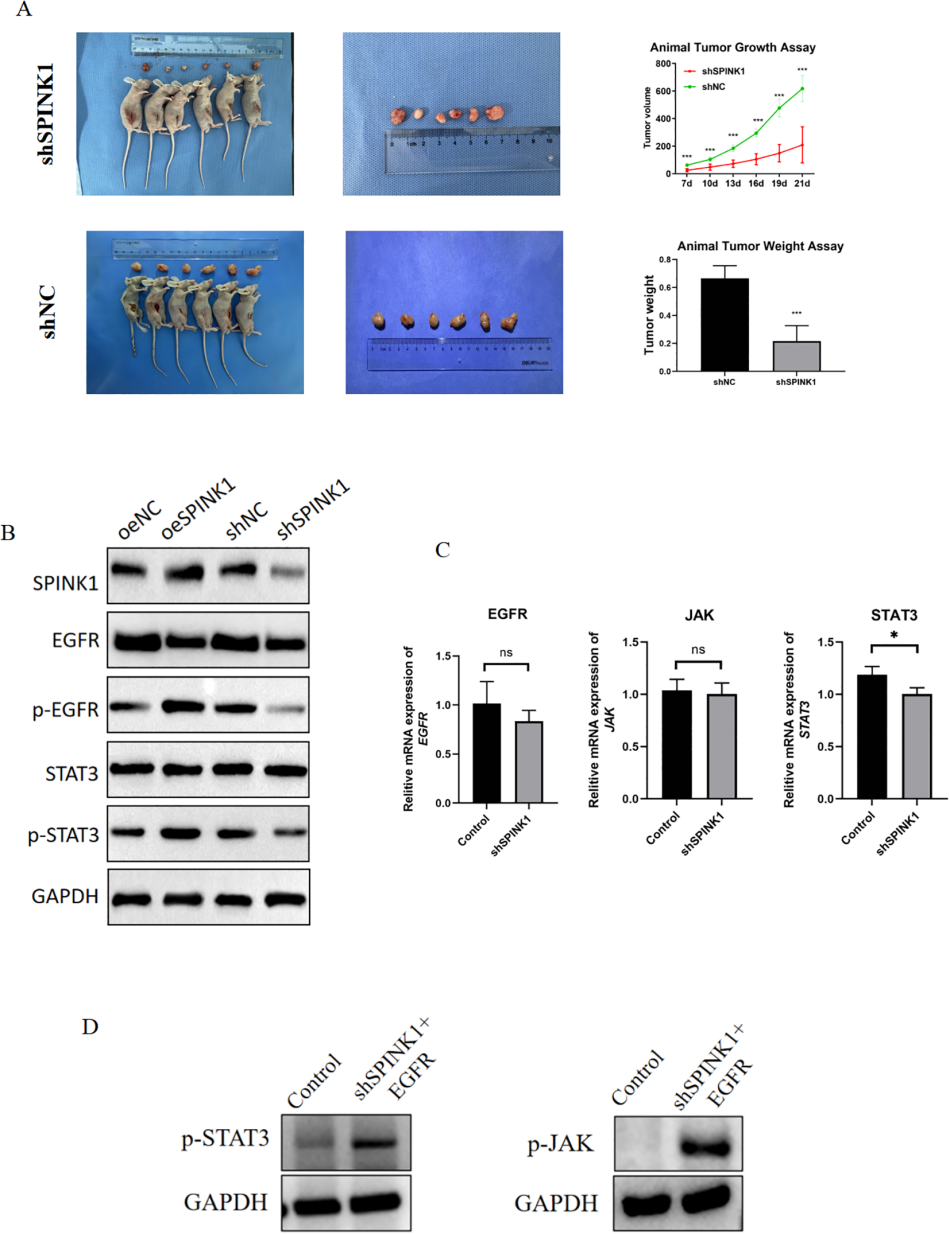
**(A)** Xenograft tumor experiments with SPINK1 knockdown; **(B)** Effects of SPINK1 knockdown and overexpression on EGFR, p-EGFR, STAT3, and p-STAT3 proteins; **(C)** Effects of SPINK1 knockdown and overexpression on mRNA expression of EGFR, JAK, and STAT3; **(D)** Effects of exogenous EGFR addition on phosphorylation of JAK and STAT3.

Xenograft models demonstrated that SPINK1 knockdown markedly inhibited tumor growth, with a 50% reduction in tumor weight compared to controls at 21 days (**Figure 5A**), underscoring its therapeutic potential.

### 3.10 SPINK1 activates EGFR/JAK/STAT3 signaling

Mechanistically, Overexpression of SPINK1 in HN4 cells significantly enhanced EGFR and STAT3 phosphorylation levels, whereas knockdown of SPINK1 in CAL27 cells suppressed EGFR and STAT3 phosphorylation (**Figure 5B**). Notably, the mRNA expression levels of EGFR, JAK, and STAT3 showed no significant changes after SPINK1 knockdown (**Figure 5C**).

To confirm that SPINK1 regulates the JAK/STAT3 signaling axis through an EGFR-dependent mechanism, we performed rescue experiments by treating SPINK1-knockdown CAL27 cells with recombinant human EGFR (20 ng/mL). The results demonstrated that exogenous supplementation of EGFR restored the protein levels of p-JAK and p-STAT3 (**Figure 5D**). These data provide conclusive evidence that SPINK1 activates the JAK/STAT3 signaling pathway in an EGFR-dependent manner.

## 4 Discussion

The present study elucidates the oncogenic role of SPINK1 in OSCC through integrated analysis of single-cell sequencing data from the GEO database (GSE215403) and experimental validation. Our findings highlight SPINK1 as a critical regulator of tumor progression and immune modulation, providing novel insights into OSCC pathogenesis and potential therapeutic strategies.

Single-cell clustering demonstrates that SPINK1-high malignant cells dominate the OSCC ecosystem, consistent with previous reports that malignant ductal cells in primary pancreatic ductal adenocarcinoma (PDAC) exhibit specific immunometabolic activities through single-cell sequencing, and SPINK1 also serves as a prognostic marker for PDAC (39). Similarly, in hepatocellular carcinoma single-cell sequencing studies, SPINK1 enhances tumor chemoresistance (40). Single-cell analysis identified SPINK1-high malignant cells with enhanced activity in chemokine signaling, Jak-STAT, and osteoclast differentiation pathways, while SPINK1-low cells were enriched in MAPK and TNF pathways. The interaction between SPINK1 and the JAK-STAT3 pathway has also been observed in both ovarian cancer and colorectal cancer (29, 30). Notably, in colorectal cancer, SPINK1 has been shown to activate the EGFR/MAPK signaling pathway, thereby promoting cancer progression, which may be related to the tissue-specific origin characteristics of the tumor (41).

GSVA analysis revealed significant enrichment of SPINK1 in canonical oncogenic pathways, including NOTCH, IL6/JAK/STAT3 and WNT/β-catenin signaling, with strong correlations to key molecules such as STAT3, NOTCH1, MAPK1, and EGFR. Mechanistically, JAK/STAT3 acts downstream of EGFR, where STAT3—as a transcription factor—directly regulates effector molecules of the NOTCH1 and WNT/β-catenin pathways, serving as a signaling hub (42–44). Notably, EGFR is one of the most critical oncogenic drivers in head and neck tumors, with several targeted therapies already in clinical use (45).

Previous studies have demonstrated that SPINK1 acts as a critical mediator in chemoresistance. In hepatocellular carcinoma, cisplatin and 5-fluorouracil treatment enhance SPINK1 transcriptional activity and drive chemoresistance via the EGFR-ERK-CDK4/6-E2F2 axis (40, 46). In pancreatic cancer, SPINK1 overexpression leads to gemcitabine resistance (47). Crucially, our study positions SPINK1 upstream of the EGFR/JAK/STAT3 pathway. In OSCC, hyperactivation of the EGFR/JAK/STAT3 signaling pathway promotes cell survival and drug efflux (48–50). Consequently, our findings provide two viable strategies to overcome cisplatin resistance: first, direct inhibition of SPINK1 using monoclonal antibodies or siRNA to disrupt its downstream signaling cascade; second, pharmacological blockade of the EGFR/JAK/STAT3 axis in SPINK1-high OSCC. Both approaches may resensitize tumor cells, representing promising targets for SPINK1 in combinatorial chemotherapy. However, this hypothesis requires rigorous validation in preclinical models.

ssGSEA further demonstrated hyperactivation of amino acid metabolism pathways in SPINK1-high cells, aligning with EGFR/mTORC1 axis-driven metabolic reprogramming. EGFR activates mTORC1 via the PI3K/AKT pathway, upregulating glutaminase (GLS) to fuel nucleotide synthesis, while STAT3-mediated cell cycle progression creates a self-reinforcing loop that provides both biosynthetic precursors and proliferative signals for rapid tumor growth (51, 52). Subsequent experiments confirmed the SPINK1-EGFR/JAK/STAT3 axis, consistent with reports that SPINK1 induces phosphorylation of downstream effectors like AKT/ERK to promote malignant progression in solid tumors (28, 53). Intriguingly, although SPINK1 knockdown did not significantly reduce EGFR/STAT3 mRNA levels, the observed downward trend suggests a potential positive feedback loop between SPINK1 and EGFR or other parallel regulators. This implies that SPINK1 may modulate EGFR activity not only through post-translational modifications but also via indirect transcriptional or translational regulation—a hypothesis requiring further validation.

SPINK1 correlated strongly with immunomodulatory factors, including chemokines (CCL3, CXCL8), immune checkpoints (ADORA2A, TGFB1), and MHC molecules (HLA-C). These findings suggest SPINK1’s dual role in recruiting immunosuppressive cells (e.g., Tregs, M2 macrophages) and evading immune surveillance, akin to its role in hepatocellular carcinoma (54, 55). In SPINK1-knockdown xenograft tumor models, a reduction in tumor weight exceeding 50% was observed. SPINK1 has also demonstrated tumor growth suppression in colorectal cancer and prostate cancer murine models (56, 57). These findings underscore the therapeutic potential of SPINK1. A promising strategy involves neutralizing its extracellular effects without altering intracellular signaling pathways through monoclonal antibody-mediated targeting. This approach merits further investigation in OSCC. Experimentally, SPINK1 was upregulated in OSCC cell lines (SCC9, SCC25, CAL27, HN4, HN30) compared to HOK, consistent with its oncogenic role in other cancers, such as non-small cell lung cancer (MEK/ERK activation)and hepatocellular carcinoma (Wnt/β-catenin signaling) (22, 56). Functional assays confirmed that SPINK1 knockdown suppressed proliferation, migration, and invasion in CAL27 cells, while its overexpression exacerbated these phenotypes in HN4 cells. These results align with SPINK1’s reported roles in promoting EMT (41).

While our study leveraged robust single-cell datasets from GEO, the limited sample size (n=12) may constrain the generalizability of the findings. Future investigations should validate these observations in larger cohorts and through spatial transcriptomics approaches. Furthermore, although this work establishes SPINK1-mediated tumorigenesis and progression via the EGFR/JAK/STAT3 signaling axis, the precise molecular mechanisms governing EGFR membrane localization remain uncharacterized and warrant further exploration.

In summary, SPINK1 drives OSCC progression via GEO-STAT3 activation and immune remodeling. Its prognostic and therapeutic value warrants further investigation, particularly in combination with EGFR or immune checkpoint inhibitors. This study underscores the utility of GEO-derived single-cell data in uncovering oncogenic mechanisms and advancing precision oncology.

## Data Availability

The datasets presented in this study can be found in online repositories. The names of the repository/repositories and accession number(s) can be found in the article/**Supplementary Material**.
