# Supplementary figures and images for: SPINK1 facilitates tumor progression via the EGFR/JAK/STAT3 axis in oral squamous cell carcinoma: insights from single-cell RNA sequencing
Source: Front Oncol. 2025 Aug 19;15:1585277. doi: 10.3389/fonc.2025.1585277 (PMC12401992; doi:10.3389/fonc.2025.1585277)

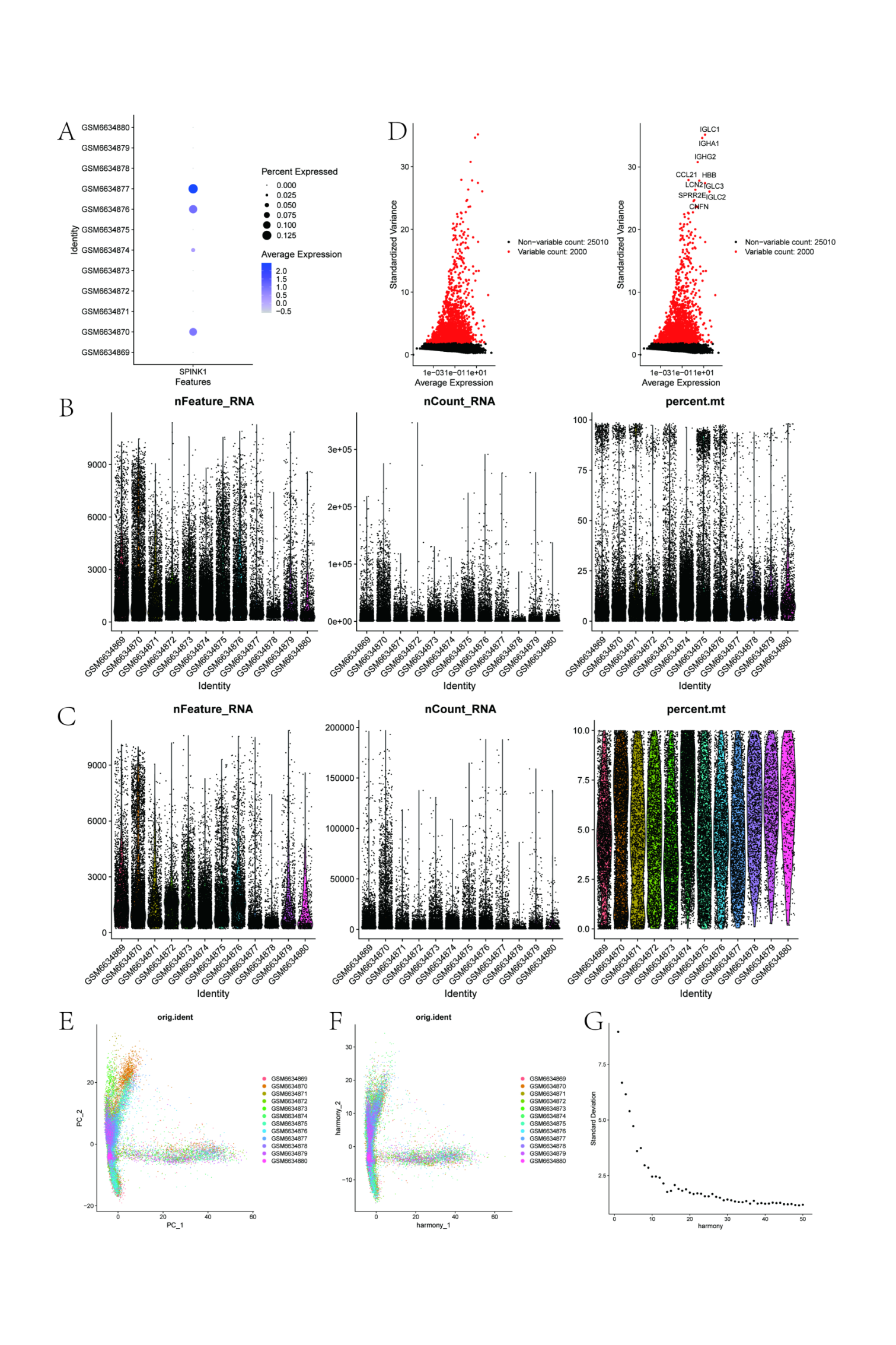

Supplement: Supplementary file 1 [file Image1.tif]
